# Mirror, Peephole and Video – The Role of Contiguity in Children’s Perception of Reference in Iconic Signs

**DOI:** 10.3389/fpsyg.2020.01622

**Published:** 2020-07-14

**Authors:** Sara Lenninger, Tomas Persson, Joost van de Weijer, Göran Sonesson

**Affiliations:** ^1^Centre for Languages and Literature, Cognitive Semiotics, Lund University, Lund, Sweden; ^2^Department of Early Childhood Education, Kristianstad University, Kristianstad, Sweden; ^3^Department of Philosophy, Cognitive Science, Lund University, Lund, Sweden; ^4^Lund University Humanities Lab, Lund, Sweden

**Keywords:** contiguity, children, sign use, indexicality, semiotic resource, visual iconic media, mirror, video

## Abstract

The present study looked at the extent to which 2-year-old children benefited from information conveyed by viewing a hiding event through an opening in a cardboard screen, seeing it as live video, as pre-recorded video, or by way of a mirror. Being encouraged to find the hidden object by selecting one out of two cups, the children successfully picked the baited cup significantly more often when they had viewed the hiding through the opening, or in live video, than when they viewed it in pre-recorded video, or by way of a mirror. All conditions rely on the perception of similarity. The study suggests, however, that contiguity – i.e., the perception of temporal and physical closeness between events – rather than similarity is the principal factor accounting for the results.

## Introduction

Pictures and film are multifaceted objects that are used by adults to further the enjoyment and education of children from an early age on. In relation to the visual meaning that is conveyed, pictures appear to be self-explanatory, even “tautological” (Barthes, 1964) and, thus, easy to understand. At the same time, they are rich in meaning, allowing for different interpretations, and act as complex communicative devices – all which contribute to making the comprehension of pictures anything but straightforward. The reason pictorial meaning is assumed to be self-evident derives from its iconic character, i.e., that they show considerable similarity with that which is depicted. An essential facilitator for the interpretation of pictorial semiotic resources is that they rely on how visual meaning is structured in everyday perception. In other words, a picture which in some sense is “realistic,” possesses traits of meaning that are intuitively paired (associated) with experiences also found in the ordinary perception of the visual world we live in Gibson (1979, 1983).

Therefore, even perceivers who are less familiar with pictures, such as infants and some animals, can recognize a visually familiar object in a picture display without having former training in picture perception (Hochberg and Brooks, 1962; DeLoache et al., 1979; Persson, 2008; Fagot et al., 2010). Indeed, previous studies indicate that very young children extract implicit and perceptually adequate meanings from realistic still pictures (DeLoache et al., 1979; Barrera and Maurer, 1981) film (Murray and Trevarthen, 1985; Marian et al., 1996; Troseth, 2010) and mirror images (Loveland, 1986). However, while several studies pinpoint the presence of an adequate appreciation of perceptual meanings in pictures from the very first months of life, other studies have demonstrated ambiguities in understanding the picture as an information resource pertaining to actual space “outside” of the pictorial medium in children up to 5 years of age (Beilin and Pearlman, 1991; DeLoache and Burns, 1994; Robinson et al., 1994; Callaghan, 2000; Zack et al., 2009).

The ability to identify an object or scene from a picture, however, is only part of what it means to understand a picture. Indeed, while a similarity relation, i.e., iconicity, is essential for something to be a picture in the first place, this similarity is nevertheless not the exclusive focus of the communicated meaning. Pictures are used to communicate information about things or thoughts that can be said to be extrinsic to the picture. A growing number of studies have taken stock of this fact, observing that the recognition of the similarity between two items is not co-extensive with understanding that one of these items is a sign for the other rather than the reverse (Sonesson, 1989; DeLoache and Burns, 1994; DeLoache, 2000; Persson, 2008; Troseth, 2010; Lenninger, 2012). Henceforth, we will use the notion of *sign* to characterize a kind of meaning relation in which one instance is directly experienced, e.g., perceived (an expression), while another instance is taken to be the focus of interest (a content). It is in this sense that we say that, from the point of view of the perceiver, a sign expression is *differentiated* from its content and/or referent (Sonesson and Lenninger, 2015). Therefore, what is a sign for one individual does not have to be so for another. In this respect, young children do not have to experience a sign relation between a picture and its object in cases in which adults customarily do so, although they can often identify familiar objects in pictures and film (Lenninger, 2012; Sonesson and Lenninger, 2015)^1^.

As noted above, previous studies have shown that children recognize similarity between objects and the corresponding pictures before they understand that pictures are used as signs (DeLoache and Burns, 1994; Liben, 1999; DeLoache, 2004; Jolley, 2010). That is, the perception of similarity precedes the understanding of how to use this relationship for specifying referential meaning. Thus, for instance, children’s experience of similarity may be neutral between seeing a picture of a doll as a sign for a doll, or just an (atypical) instance of the category of dolls (DeLoache et al., 1979). Moreover, young children have been observed to imitate video-transmitted actions. Provided that the actions are adapted to the age of the children, 14- and 15-month-old children imitate new actions on objects demonstrated in pre-recorded video (Meltzoff, 1988; Barr and Hayne, 1999). Responsiveness to video in terms of imitation of actions does, however, not necessarily require detection of sign relation, only mapping of behavior (Hribar et al., 2014; Troseth et al., 2019). In addition, a video deficit effect in young children’s learning from video demonstrations compared to learning from in real life perception (Anderson and Pempek, 2005; Schmidt et al., 2007; Suddendorf et al., 2007; Troseth, 2010) suggests a media related difficulty in transferring meaning from video.

Pictures are about more than iconicity. They are often employed to realize certain functions, for instance to inform about circumstances or states of affairs in the world outside of the picture, as when they are employed to set the goal of searching for a hidden object (DeLoache and Burns, 1994) or for an object which is in plain view in another room (Lenninger, 2012) or to instruct the subject about a box in which an interesting object is hidden (Potì and Saporiti, 2010). In these examples it is not just the perceptual similarity between a medium and what it depicts that makes something into a usable piece of information about the external world. When it comes to children’s ability to utilize information from visual media in real-world problem-solving tasks, three aspects in particular have been investigated, besides age differences. Two of these are the accuracy of perceptual similarity (iconicity) in the media, and the use of language in relation to the picture (DeLoache et al., 1999; Callaghan et al., 2012). Both these conditions remain influential in young children’s learning from media also when a third condition is investigated: the manipulation of contingency, that is, a perception of responsiveness in the interactions with iconic media (Troseth et al., 2019).

Especially the youngest children have been shown to be susceptible to the level of perceptual accuracy in pictorial presentations (Callaghan, 2000; Simcock and DeLoache, 2006). Notably, when both media are accompanied with equivalent narrative support, 18 months young children are more successful when it comes to re-enacting a novel action sequence from video instructions as compared to picture books with realistic pictures (Simcock et al., 2011). Moreover, in the same study, children managed to imitate actions from a verbally based narration alone, without the presence of any pictures at all. Thus, it seems that the rich iconic information present in video, displaying the full sequences and movements instructively, supports young children’s re-enactments, while it is less clear whether information via still pictures do so for tasks that require physical actions. In a different research paradigm, however, investigating young children’s word-learning in a training procedure with pictures, iconicity has been shown to make a significant difference for the younger perceivers. Studies have shown that even as young as 15 months old children can extend names learned from naming photographs and realistic drawings, while not benefitting from the same procedure using cartoons, suggesting that perceptual realism carry weight when it comes to transferring newly learned words from pictures to their corresponding objects (Ganea et al., 2008). Interestingly, in another study of learning words via pictures, it was found that perceptual similarity had an effect on 30-months old children in the sense that words learned from sketches tended to remain meaningful only for a sketched version of that object, while words learned from photographs to a greater extent were extended also to the corresponding physical objects (Mareovich and Peralta, 2015).

Children’s contingent or non-contingent experiences using pictorial media have been studied in two major paradigms: social contingency (de Saussure, 1968; Strouse et al., 2018) and physical contingency (Lauricella et al., 2010; Choi and Krikorian, 2016; Jauck and Peralta, 2019). Social contingency, when children respond according to social cues in their interaction with pictorial media, has been investigated in two directions. First, social contingency can work in the direction toward the medium – such as when parents take part as co-viewers and support 2-year-old children in learning novel verbs from video (Strouse and Troseth, 2014). Second, a social partner provided by the media, the perception of social-emotional content in media, and child-directed communication in the media all have been suggested to facilitate young children’s learning from video interactions (Fenstermacher et al., 2010; Dayanim and Namy, 2015; Troseth et al., 2019). While both on-screen social contingency and co-viewer’s support help children to learn from video, the in-person co-viewer seems to be the most supportive of the two (Strouse et al., 2018). Physical contingency has been suggested to promote children’s perception of reference meaning (Kirkorian et al., 2016) and is established by the perception of a responsiveness from the media related to one’s actions toward the medium (Troseth et al., 2019). This experience of meaningful responsiveness can occur for the child when scrolling and clicking on a touch screen, tilting the tablet in order to slide a figure to a new position in a game, or swipe the surface and change the interface etc. (Lauricella et al., 2010; Choi and Krikorian, 2016). The experience of physical responsiveness in visual media can also occur in combination with the help of a social partner, such as when a parent points out a relationship between the child’s play in front of the video camera and the live video projection on the television at home (Troseth, 2003).

Another factor of importance for understanding semiotic resources (i.e., the resources for meaning-making, e.g., pictures or video), we will suggest, is the perception of indexicality, prevalent in experience of the lived world, and the rupture introduced to this contiguity following the use of certain media. This links to the possibility that the semiotic resources can be understood by means of the spatial situatedness of the semiotic resource at hand, in relation to the perceiver and the referent. Meaning-making thus also involves indexicality, including in the sense of contiguity (Peirce, 1931–1958; Piaget, 1945; Bruner, 1966; Bates et al., 1979). Indexicality and iconicity are not independent of each other. The iconical character (the way it conveys similarity) of the semiotic resource may sometime be involved in determining its indexicality, and vice-versa. For example, a mirror image has to occupy a different position in relation to the perceiver and the referent than the other resources (indexicality), which changes the image that reaches the perceiver (iconicity). Because of the interactions of indexicality and iconicity, only what we will call *higher order iconicity* (i.e., a corresponding event involving the same constellation of objects) can be expected to be held constant between treatments. Our hypothesis is that indexicality has an impact on the perception of information about the external world, also in the case of media that are predominantly based on iconic relationships, and, irrespectively, of the perceiver having developed the habit to use sign relations or not. Indeed, it will be suggested that, in the particular case studied in the following, indexicality accounts at least as much for the results as iconicity.

### Variation of Iconicity in Retrieval Games

Finding a hidden object by means of visual information in a picture or a film showing the hiding place requires using visual information to guide action (search) in a way that is not mere imitation (DeLoache, 2004). DeLoache and Burns (1994) found that 2-year-old children can retrieve a toy from a hiding place in an adjacent room if they are told where it is. If, on the other hand, they are shown a realistic picture (i.e., a photograph or a realistic drawing) indicating where to find the toy, one cannot expect them to retrieve it, in spite of the fact that children are customarily raised with pictures being part of their daily social environment. Six months later, however, children can typically use pictures to guide their search for hidden items – even if it is the first time that they are using pictures in this way. Akin to the *object retrieval games* described above, young children’s understanding of scale models or video as sources of information has also been tested (DeLoache, 1987; Troseth and DeLoache, 1998; DeLoache, 2000; Schmidt and Anderson, 2002). In sum, these studies indicate that at the age of 30 months children can use pictures and video recordings as sources of information about the world. Children achieve corresponding results with scale models around the age of 36 months. Thus, the three-dimensionality in the scale model, compared to the two-dimensionality of the picture, appears to be an iconic feature that does *not* facilitate retrieval. Intriguingly, when the medium is made “transparent,” such as when a video clip can be perceived as being the real scene seen through a window, or when the scale model is mistaken for the actual, magically size-reduced space, children manage to retrieve the toy already at the age of approximately 24 months (DeLoache et al., 1997; Troseth and DeLoache, 1998; Lauricella et al., 2010). Hence, although the visual information is nearly the same (except for size), children tend to alter their appraisal of the video and scale-model depending on whether they perceive them as reality or not (Troseth and DeLoache, 1998). A possible reason for this difference is the reduced need for having to ascribe a sign relation to the visual information afforded when it is not taken to involve any specific medium (DeLoache et al., 1997).

Interestingly, the salience (DeLoache, 2000) of the medium itself is an aspect of iconicity in iconic media. Hence, while the aforementioned results indicate that the *suppression* of distinctive features of the media can render their function as an information source more accessible, the opposite manipulation, which could be interpreted as *enhancing* the media and making the sign expression appear more unreal (and thus reduce iconicity), has also been shown to improve the performance among young children. When a scale model was put behind a clear plastic window, thus making it less accessible to interaction, the youngest children (24 months of age) improved their search of the real room (DeLoache, 2000). While this procedure does not eliminate the perception of three-dimensionality, in the sense of impeding the functioning of the motion parallax and binocular disparity cues, the scale model was certainly made more “picture-like,” simply by being framed, and perhaps more importantly by manipulation being rendered impossible. This could be sufficient to increase the need for the ascription of a sign relation to the relation between the objects, in order to link them meaningfully to the outer world.

Everything considered, although iconicity (i.e., the potential to be similar) is a variable that matters in object retrieval games, altering iconicity is not a straightforward parameter for manipulating children’s success in retrieving a toy. Moreover, the suppression or accentuation of media characteristics, amounting to a modification of sign relations, clearly plays a part, as shown in some of the studies referred to above. The present study is involved with three varieties of iconicity in (potentially) temporally continuous visual signs, that is, in live streaming video clips, pre-recorded video clips, and mirror images. These meanings are compared to the direct perceptual experience of the same event. While all four kinds of visual meaning share some amount of iconicity, intuitively there is a sense in which the three kinds of signs are iconically different from direct perception of the actual world. In addition, the two kinds of video clips share more iconic properties with each other than either of them with the mirror image. Nevertheless, all four ways of conveying the information are identical, from the point of view of what we call higher order iconicity: they convey the same event. Moreover, all conditions rendered an identical visual framing of the scene (i.e., the cups and the torso of the person who hides the tokens), showing the hiding event with the similar cups and tokens, all from a frontal perspective. The two kinds of video share a kind of *lower order iconicity*, in that they apply the same filter to the event, i.e., their perceived video quality, while mirror images manifest another kind of lower order iconic specificity, their perceived mirror character.

### The Factor of Indexicality in the Present Study

In this study, we suggest that the perception of iconic *and* indexical relations is vital for creating meaning in everyday experiences (Michotte, 1941; Piaget, 1947; Bates et al., 1979; Gibson, 1983; Oakes and Kannass, 1999; Rickardson and Kirkham, 2004; Merleau-Ponty, 2012). Indexical meaning is here understood as the perception or expectation of contiguity. In other words, in indexical relations, parts are presumed to be in proximate or direct connection to each other in *time*, *space*, or both. Hence, in ordinary perception, two sides of an object that look very different from each other are conceived as, and expected to be, part of the same object. Moreover, in an environment physical space is expected to continue around a corner, even though it is occluded for the perceiver, and for instance, a perceived body part sticking out from the corner is expected to be part of a full body (Piaget, 1947; Gibson, 1979).

Contiguity can be a cardinal factor in establishing meaning even when similarity is the predominant factor in the meaning relation. Consider, for example, the significance of contiguity in the proximate relation of one’s face to its mirror image, or for the actions motivated by the rear-view mirror in driving a car, or in the information from a surveillance camera. Contiguity is indeed a factor that can vary in different semiotic resources which otherwise share an iconic ground. Our aim is not to downplay the importance of achieving reference by means of the sign relation – but to study the role of indexicality in terms of the perception of contiguity as a factor present in media that are primarily based on meaning conveyed by means of an iconic ground. It is for this reason that we contrast 2-year-old children’s understanding of three related visual iconic resources: live video, pre-recorded video and mirror images.

We want to stress three points in support of this choice. First, live video, pre-recorded video and mirror images all establish meaning by constituting visual similarities (i.e., they are all based on perceptual iconic grounds). Second, however, they differ with respect to conditions that make it possible to keep track of a contiguity to their reference objects in the here-and-now. Third, we do not expect children 2 years of age to grasp the sign relation in pictures; that is, we do not expect them to link a pictorial expression and a referent as being two related – but separate – instances of visual meanings. Hence, although 2-year-old children are expected to be familiar with watching video or television, and although they may well perceive a video projection as something different from reality experienced here and now, they do not necessarily use the abstract sign relation in order to link the two instances of meaning (Piaget, 1945; DeLoache and Burns, 1994; Troseth and DeLoache, 1998; Liben, 1999; Pierroutsakos and DeLoache, 2003). On the other hand, if perception of contiguity facilitates the linking of one instance of meaning to another (independently of adopting meaning by way of a sign relation), then conditions that support the perception of contiguity connecting a hiding event to a retrieval event should be more beneficial in this age group than conditions that clearly prevent perception of such contiguity.

### The Present Study

In the experiment, 2-year-old children watched an attractive object being hidden under one of two cups. They witnessed this event in one of the following four conditions: directly through an opening in a cardboard screen, by means of live video on a computer monitor, by means of pre-recorded video on a computer monitor, or in a mirror. The children were then presented with the two cups they had seen and were stimulated to search for the object under one of them. According to our underlying assumption, these conditions pose different problems with regards to the way of employing the different semiotic resources for making the choices. Specifically, the general aim of the study was to assess contiguity as a variable for connecting visual meanings separated in time and space, independently of sign function. In the experimental situation, the children encountered two different categories of objects, that was, the cups in the video and the actual cups at the retrieval place, and yet it was hypothesized that, under certain circumstances, these objects could be perceived as being contiguous and thus potentially spatially and temporally continuous. Such media as allow for a presumed continuous perception of the target cup witnessed at the place of hiding and of its transition to the place where choices had to be made, may potentially facilitate the children’s access to the information needed for making correct choices. Perception of contiguity was expected to be afforded by live video and in the mirror condition. In live video and in the mirror trials the actual hiding events occurred at the same moment in time, though in appearance not in space, as the event exposed for viewing, just as in the direct perception.

**FIGURE 1:**
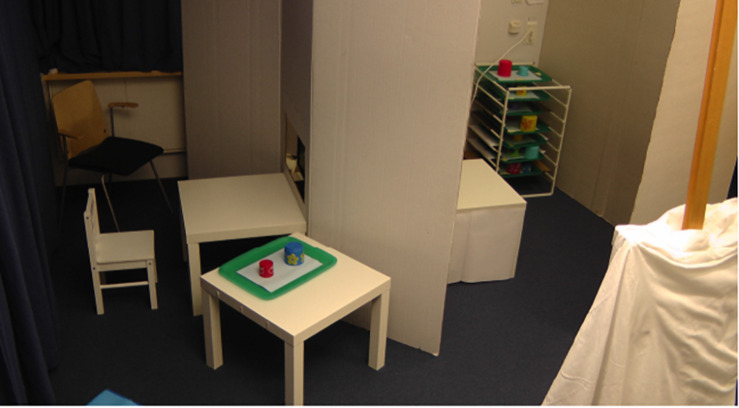
The experiment space in the position for making a choice between the two cups. The mirror, which is covered here, is visible in the lower right corner of the image and the prepared trays (used only for recorded video) are seen in the back of the experimenter’s space.

**FIGURE 2:**
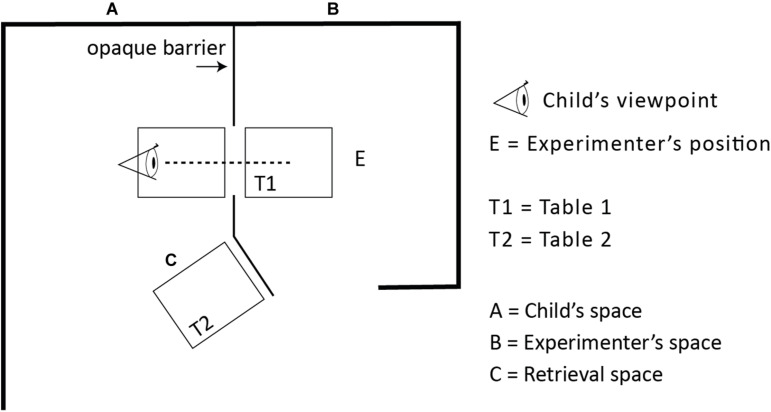
The figure illustrates the direct perception setup with direct view of the hiding event through an opening in the barrier. The location was divided by a cardboard occluder (the barrier) into the child’s action space **(A)** and the experimenter’s information space **(B)**. At **C** the experimenter and child met, and the child made her choice in selecting a target cup from the tray that was brought to T2 by the experimenter. In the trials with video (recorded video and live video) a monitor was mounted in the opening (with exact fit) so that the direct view to E was prevented.

Maintaining clearly separated places for information and retrieval was crucial for the design of the test, since such a discontinuity demands a bridging operation in the form of perceiving contiguity, or a sign relation. The two places were expected to be clearly separate to the child, and therefore subjectively experienced as two different places and not confused as one and the same. At the same time, the two places were both within the child’s perceptual reach during the entire session. Moreover, although the two places were proximate, the child had to take a few steps and face different directions in the shifting of places (see Figure 1). The experimenter’s moving the cups from the place where the hiding was perceived (see T1, Figure 2) to the place for the retrieval (see T2, Figure 2) further enhanced the separation of the two locations. With these arrangements we aimed to lower the risk of the children misperceiving the locations and thereby missing the discontinuity between the “media-cups” and the “reality-cups.”

Note also that the children never entered the experimenter’s space (B in Figure 2) where the actual hiding events were perceived or conceived. This is an important difference to other studies in which object hiding and retrieval were not separated in space (Menzel et al., 1978; DeLoache and Burns, 1994; Troseth and DeLoache, 1998; Suddendorf, 2003; Lauricella et al., 2010). It is also a difference from the experimental design of the Potì and Saporiti (2010) study involving capuchin monkeys, which made use of a two-place design, but did in no way permit the observation of the displacement of the cups from one place to another, thus making it possible, and indeed probable, for the monkeys to confuse the two places.

One of our premises was that, in this context, mere iconicity was relatively unproblematic. As suggested above, the different ways of conveying information about an event are iconically identical at the higher order level (i.e., recognizing the same basic event in the different conditions) and the variations at the lower order levels of iconicity (i.e., the media-specific filtering of the basic event) was equal in live and pre-recorded video (filmed by the same camera projected on the same screen) and as equal as possible in the mirror reflection (perceptual size and form of the mirror). Therefore, we expected the children watching the video or the mirror to be able to perceive under which cup the token was being hidden (Murray and Trevarthen, 1985; Loveland, 1986; Barr and Hayne, 1999). In addition, we expected 2-years-old children to distinguish video and mirror images from ordinary perception (Liben, 1999; Troseth, 2010). It should be noted, however, that we did *not* expect 2-years-old children to have recourse to sign meanings in their perception of pictures, videos or mirrors (DeLoache and Burns, 1994; Troseth and DeLoache, 1998; Allen et al., 2010; Sonesson and Lenninger, 2015). The task set for the children in this game was rather to guide their object choice by connecting the perceived information as it was conveyed by the different media to real-world objects, in the absence of sign relations. In this age group children are not expected to fully have appropriated the differentiated sign relation (Piaget, 1945; DeLoache, 2004).

The prediction was that the condition in which information stemmed from direct perception would be the easiest to master and consequently would generate most successful trials. Secondly, we expected performance in live video to be second best since it affords cues for contiguity close to those in direct perception. In contrast, the hiding and retrieval events in pre-recorded video are discontinuous, and thus they were predicted to be difficult for the children. Previous studies indicate that 2-year-old children are more successful in gaining information from live video than from pre-recorded video. In a retrieval game, children were shown to have more success finding the hidden toy when informed by an in-the-moment responsive on-screen person in a live video than from an on-screen person in a pre-recorded film, although this person also acted in a social child-friendly way (Troseth et al., 2006, 2019). In the present study, however, none of the conditions involved interactive chat communication between the perceivers and the person in the video. This is because the study was designed to measure difference in terms of perception of contiguity.

We did not have a specific expectation for the mirror image. The mirror image is distinct from ordinary perception. Even though the viewing angle can be subjectively adjusted in mirror perception, the continuity of the mirror image to the corresponding scene is disrupted (cf. the hiding event in this study). The mirror displays its object (cf. the hiding information) from a different direction than the place where the scene or act occurs in the real room. Moreover, the enantiomorphic nature of the mirror image implies a reversal of the directions within the scene. However, there have so far been too few studies about how 2-year-old children understand the mirror image as a source for visual information for any conclusion to be drawn. In a different research paradigm, following Gallup (1970) mark test, designed to indicate self-recognition, it has repeatedly been shown that 2-year-old children perform self-directed actions when presented with their own mirror images. The assumption is that self-directed behaviors when presented with their mirror image indicate the emergence of self-awareness (Gallup, 1970; Amsterdam, 1972; Asendorpf et al., 1996), or, alternatively, that it demonstrates an ability to cope with multiple senses of selves (Povinelli et al., 1996). Since the mark test is accomplished more or less at the same time as children have success in retrieving a depicted object in the real world, one may think that what is at stake in both cases is the discovery of the sign function of the picture (Sonesson and Lenninger, 2015). In fact, whether or not the mark test indicates self-awareness, it does not necessarily indicate an understanding of the mirror image as a sign. Due to the physical contingency provided by the mirror reflection, kinaesthetic-visual-matching may be enough to guide behavior toward an unexpected mark on one’s face (Mitchell, 1997). Moreover, studies designed to investigate transgressive behavior have shown that the presence of a mirror serves to adjust the behavior of 3-year-old but not that of 2-year-old children (Martin et al., 2019). In the present study the mirror was directed to reflect the place of the hiding event, but not the body of the perceiving child.

## Materials and Methods

### Method

#### Participants

A total of 36 children participated, 22 of which were girls and 14 boys. Their mean age was 24 months and 18 days (ranging 23–25 months, SD = 12 days). They had been raised in Sweden and had no known developmental disorders. Caregivers reported normal visual acuity. The children were recruited from birth records and were randomly chosen from an address pool with caregivers who had agreed on being contacted for the purpose of the study. All participants lived in the south of Sweden at the time of the study.

### Materials and Setup

**FIGURE 3:**
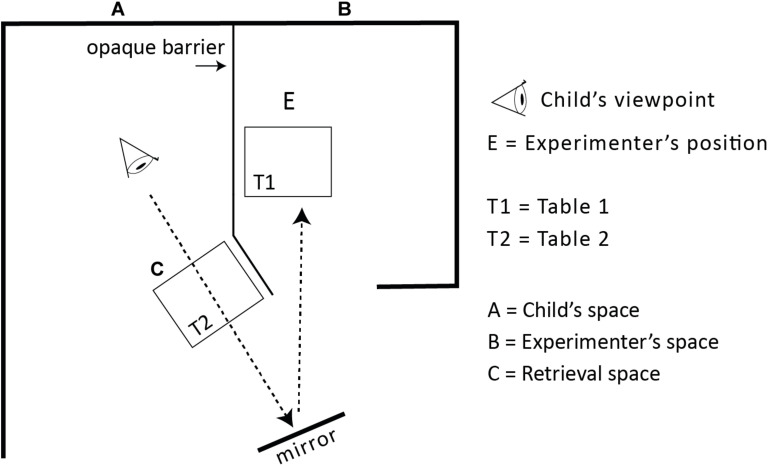
The figure illustrates the setup for the mirror trials. When informed via the mirror the opening in the barrier was closed so that the direct view to E was prevented. The mirror was placed so that the child could peek around the corner and perceive the hiding event through the mirror. In these trials the experimenter changed position at T1(B) to face the child’s viewing angle in the mirror.

The experimental setup is illustrated in Figures 1–3. The experimental room was divided into two spaces (one for the child, the other for the experimenter) separated by a 185 cm high cardboard screen. Child-sized tables were positioned at both sides of the screen, as shown in Figure 2. On the child’s right side, only a few steps away and always in sight from the child’s viewing position, a second child sized table was placed (T2). At this table, choice-making took place.

A 48 × 29 cm rectangular window was cut out of the screen 52 cm above the floor. The cut-out piece was used as a shutter when changing equipment between conditions in the test as described below. A mirror (40 × 255 cm) was placed on an easel (45 cm from the floor) at a distance of 215 cm from the child. A red colored light bulb attached to the top of the mirror could be turned on and off with a remote control. The mirror was covered under a white sheet when not in use (as shown in the bottom right corner of Figure 1). Moreover, three hand puppets were used as attention catchers. A box (30 × 30 × 21 cm) in which the child could put the tokens it retrieved was constructed for the study. When a token was put in the box it elicited a sound reward. Finally, 34 cups of different sizes and colors, and decorated with different patterns, were used for hiding the tokens. The tokens were made of wood with a diameter of 30 mm. Nine trays (one tray for running the warm-up phase, direct perception, live video and mirror trials, and eight trays prepared in advance for the pre-recorded trials) were placed on a tray rack.

#### Procedure

All experimental sessions started with 5 min of information and the signing of a consent form in a room separated from the test studio. Caregivers were told that they were allowed to watch the game together with their children, and say things like “look there!”, or point at the event. They were explicitly asked not to touch the screen, use words like “mirror”, “video,” “TV,” “hiding” or “cup,” or intervene in the child’s choice.

After the instructions and information had been given to the caretakers, the experimental session started with a warm-up phase to introduce the rules of the game and to familiarize the children with the sound-reward box. During this phase, a single token was placed on a tray on table T2. The experimenter immediately encouraged the child to pick up the token and to put it in the box. Subsequently, the child saw the experimenter hiding a new token under a single cup on the tray and was again encouraged to take it and put it into the box. After that, a new token was hidden under one of two cups placed next to each other. The child was encouraged to retrieve the token but was allowed to look under one cup only. After finding the token, the procedure was repeated once with the token hidden under the other cup.

**FIGURE 4:**
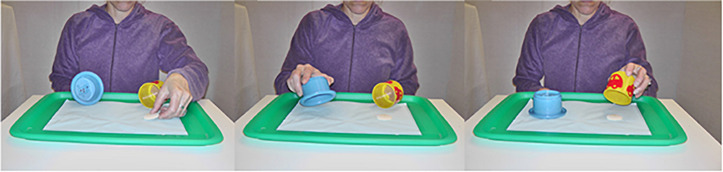
The hiding always followed the same sequences in the same order: the empty cups faced the child, the token was put in front of the correct choice, the experimenter first closed the unbaited cup, and then closed the baited cup.

After the warm-up (which all children found easy), the child and the experimenter took their places, while the caregiver sat down right behind the child on the left side. At this point, the direct perception condition began. The experimenter caught the child’s attention with a hand puppet through the opening, and then placed two cups on a tray on table T1. Subsequently, the token was put in front of one of the cups (target cup), then the non-target cup was placed upside down, and finally the target-cup was placed upside down over the token and thus covered it from sight (Figure 4). Immediately afterward, the experimenter brought the tray to T2, asking the child to come and find the token, and to put it in the box in order to elicit a sound. This procedure was repeated eight times. Left and right hiding positions were counterbalanced according to prepared semi-randomized protocols. The tokens were not hidden more than twice in the same position to avoid that children benefited from perseveration. The cups were randomly selected for each trial by picking them from one basket which contained only red and green cups, and another one with only yellow and blue ones. This routine was designed to avoid pairing green with red cups in a single trial, as we did not know whether any of the children was color blind.

Only one choice was permitted per trial. A child who did not find the token was immediately encouraged to witness a new hiding. The tray was taken back to T1, so that the child could see it being cleared. *Comfort trials* were given by way of a new hiding at T2 if the child indicated frustration or disappointment (verbally, bodily or initiating new games, running around etc.), or when the child had chosen the non-target cup three times in a row. The comfort trials did not substitute for the eight test trials. After the last direct perception trial, the shutter was closed, and a monitor was put in the opening so that the child could no longer look into the experimenter’s space.

Given the children’s age, we were worried that having them endure all four conditions during one experimental session would be too much. Therefore, each child was exposed to one of six possible combinations of two of the three remaining conditions after the direct perception condition. As a result, the direct perception condition was done by all 36 children while each of the other three conditions was done by 24 children. The hiding procedure in the other three conditions was similar to the one in the direct perception condition, except that it was viewed indirectly from a media display as described below. Also, the children were once more given eight trials, and comfort trials were offered according to the procedure described above. The first condition started directly after the last direct perception trial, the first experimental condition followed, but the children were given a 5-to-10-min break with juice refreshment and picture book reading with their caregiver, before the last experimental condition began. The entire session took 30–35 min to complete, including the break. All sessions were recorded with a Panasonic HDC-HS700 camcorder.

##### Live video

In this condition, a camcorder (Panasonic HDC-HS700) recorded the experimenter hiding the object under the cups. The camcorder was placed in the opening in the cardboard screen so that it had the approximate same viewpoint as the child. The recording was projected simultaneously to a 42-inch monitor (HP Compaq LA2205wg) which was placed in the opening of the cardboard screen and therefore blocked the view into the experimenter space. A trial started with one of the hand puppets appearing on the screen to catch the child’s attention. The experimenter’s shift from the attention-catching to the start of the hiding game was continuously visible to the child in live video. The camera was never turned off during the live video condition, and thus the child could watch the experimenter leaving and coming back.

##### Pre-recorded video

In this condition, the same 42-inch monitor was placed in the opening of the cardboard screen. Film clips, showing the hiding event, were projected on the monitor. The clips had been prepared in advance and their presentation was controlled by the experimenter from a computer placed in the experimenter’s space. Similar cups and tokens as in live video were used, and the trays had been prepared in advance in accordance with the order of the video clips. In order to emphasize that the event shown on the screen was not linked in time and space (as in live video) the stimuli were different in four aspects: (a) the attention-catching puppets were animated with sound effects, (b) the hiding event was recorded in a visibly different environment although projected from the same angle and visuospatial section (only showing the torso and the hand of the hider), (c) the hider was another person wearing other clothes than the experimenter, and (d) the monitor turned black when the hiding event was completed. As in the other conditions it was the experimenter who offered the choice between the real cups to the children.

##### Mirror

**FIGURE 5:**
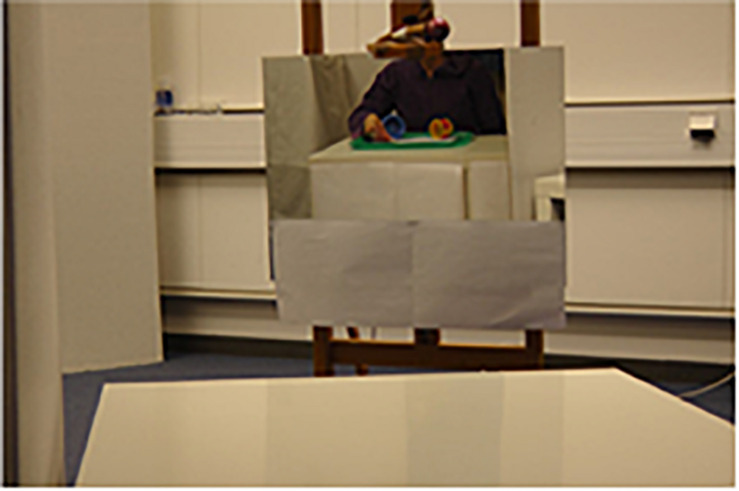
Seeing the hiding in the mirror from the child’s perspective.

In all conditions but the mirror test, a sheet covered the mirror to preclude children using it to see what was happening at the other side of the barrier. This sheet was now removed, and the mirror remained uncovered throughout the full procedure in this condition. The experimenter faced the mirror frontally, using a remote control to switch on or off the red light on top of the mirror in order to direct the child’s (and the caregiver’s) gaze toward the mirror. The hiding took place behind the screen, at table T1B, as in the other conditions. A trial started with one of the hand puppets showing up in the mirror, after which the child could see how the experimenter hid the token under one of the cups, and then headed over to table T2. Figure 5 shows the hiding in the mirror condition from the perspective of the child’s view.

## Results

All children participated with enthusiasm in the direct perception trials, and the feedback from comfort trials encouraged the children to engage in new trials in cases where the actual testing trials were experienced as being difficult or uninteresting to the child. The scores are binary, i.e., the choice was either correct (the target cup) or incorrect (another cup, or no response). One session with mirror trials was interrupted after four trials, and the remaining trials were scored as incorrect. Moreover, due to technical error, the scores from one child were replaced with the scores from a supplementing child.

Table 1 shows an overview of the results. The direct perception condition elicited more correct responses than any of the other conditions. Within the three experimental conditions, most correct responses were found in the live-video condition, and fewer in both the pre-recorded video and mirror conditions.

**TABLE 1:** Results overview.

Condition	Total trials	Correct trials	Proportion correct
Direct perception	288	221	0.77
Live video	192	127	0.66
Pre-recorded video	192	94	0.49
Mirror	192	98	0.51
Total	864	540	0.63

The response accuracy in the four conditions was analyzed as a mixed-effects logistic regression. The analysis was performed in R version 3.6.2 (R Core Team, 2019) using the packages lme4 (Bates et al., 2015) and multcomp (Hothorn et al., 2008). The analysis was performed in three steps. As the first step, we compared a model with by-child random intercepts to a model with by-child random slopes (i.e., over eight trials within each condition). If the latter model turns out to be significantly better than the former, that would be an indication that the accuracy rates increased or decreased across the eight trials. The comparison suggested that this was not the case. The difference between the two models was not significant (*X*^2^ = 0.289, *df* = 3, *p* = 0.962). In the second step of the analysis we compared the random-intercepts model selected in the first step with a model that also included condition as a predictor. This comparison was significant (*X*^2^ = 53.346, *df* = 3, *p* = 0.000) suggesting reliable differences in accuracy rates across the four conditions. In the third step of the analysis all six pairwise comparisons were tested statistically using generalized linear hypothesis testing. The results are displayed in Table 2.

**TABLE 2:** Pairwise comparisons.

Contrast	Estimate	Standard error	*t*	p*
Direct perception-live video	0.516	0.209	2.470	0.065
Direct perception-pre-recorded video	1.254	0.204	6.141	0.000
Direct perception-mirror	1.167	0.204	5.728	0.000
Live video-pre-recorded video	0.739	0.217	3.405	0.004
Live video-mirror	0.651	0.216	3.012	0.014
Pre-recorded video-mirror	−0.087	0.209	−0.417	0.975

*p-values have been adjusted for multiple comparisons.

**FIGURE 6:**
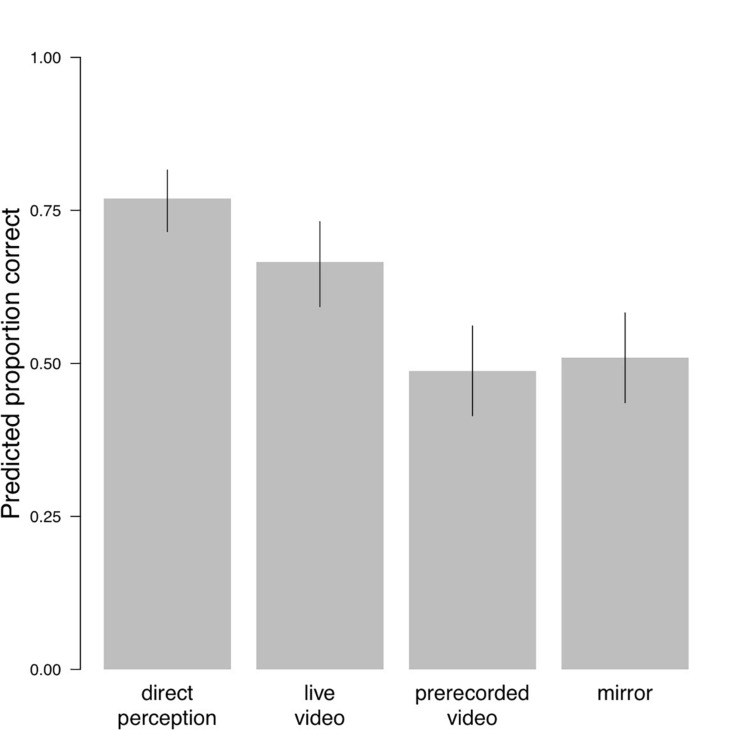
Predicted proportions correct. Error bars show 95% confidence intervals.

As the results in Table 2 show, the accuracy in the direct perception and the live video condition was significantly better than in the other two conditions. Additionally, the accuracy in the direct perception condition was marginally more significant than in the live video condition, while the difference between the pre-recorded video and the mirror condition was not significant. Figure 6 shows the expected proportions of correct answers for the four conditions together with 95% confidence intervals. The confidence intervals in the direct perception and the live video conditions do not include chance performance (*p* = 0.5), but those in the pre-recorded and mirror conditions do.

## Discussion

Meaning grounded in similarity is fundamental for what we characterize as iconic signs. *Perceptual* similarities with referent objects constitute the predominant qualities in iconic media such as pictures and video. Despite its prevalent role, however, iconicity does not have to be the single relational factor having an effect on meaning perception in iconic media. In this study we examine whether indexicality, in terms of contiguity, may play a significant role in determining young children’s ability to track information from perceptually iconic sign resources in order to solve a problem in real space. The children in the current study were most successful in choosing the target cup after having witnessed the hiding in either the direct perception condition or from watching live video. In accordance with our expectations, the number of successful choices in the direct perception condition also marginally significantly exceeded those in the live video. The scores for successful choices in pre-recorded video and mirror trials were, however, both low.

All the three tested media provide a realistic image of the visual world. Moreover, in all test conditions the hiding procedure was performed identically, allowing the child always to perceive the hiding from the same angle (i.e., from a front view). Nevertheless, the children performed significantly better when receiving the information by watching live video than by watching pre-recorded video or by way of mirror images. Contingency factors were kept as similar as possible across the tested media. The video presentation did not respond to physical interaction (i.e., it was not a touch screen) and there was no repetition of a single hiding trial. In order to avoid variations due to in-media social contingency across the media types the hider’s face was not shown in any condition and the experimenter remained silent (mute) across the hiding events in all conditions. Parents’ co-viewing followed the same instructions in all conditions. The stepwise introduction of the game in the pre-phase and base trials enabled an almost language-free training and game procedure. For the experimenter, words were only used at the retrieval place asking, “Where is the token, can you help me find the token?” and to engage in a new trial, “Shall we play it again?” Our interpretation is that variations in perception of contiguity, which was differently elicited in the trials, accounts for the difference in the probability of the children choosing the target cup.

Live video was the test condition that elicited the perception of the transition from place B to C (i.e., from the actual place of the hiding to the place of making the choice) most similar to direct perception; more so than pre-recorded video and mirror images. The mirror was the only condition where the occluder did not stand in the way for the children to keep track of the experimenter and the cups. Most obviously, mirror perception is enantiomorphic, giving rise to visual conversions to opposite forms and directions. Pre-recorded video, on the other hand, was the most discontinuous condition. Although pre-recorded video yielded visual information about the hiding of a token, it lacked informative cues to connect the video information with the retrieval of the object, because the procedure was recorded at an independent location, and there were no cues for the experimenter leaving the place which connected this event to the bringing of the cups to the retrieval place. Thus, it can be surmised that pre-recorded video provided fewer cues for a perception of contiguity than any of the other conditions. That the cups, and the location of the reward tokens, appeared identical on the screen and during the choice was apparently not enough for successful performance.

The children performed less successfully in live video than they did in the direct perception condition. This accords with our predictions, that direct perception should be the easiest condition, i.e., the condition where the children retrieved the most tokens. Only in direct perception trials were the cups presented for the child perceptually unaltered across a trial (from perceiving the hiding to making the choice). According to previous studies, transference between 2D and 3D presentations make learning difficult for young children (Zack et al., 2009; Barr, 2013). Memory flexibility (Barr, 2013) was required in all tested conditions and therefore should not account for the differences between live and pre-recorded video. We suggest, however, that the relatively frequent choices of unbaited cups in direct perception trials (almost one out of four) can be explained by the two-place design.

Some arrangements involving the different media were needed that may have affected the results. For instance, the mirror had to be adjusted to the physical spaces so that the visual frame of the hiding event was equal to the video projections (tray, cups, and experimenter’s torso, but not face). The distance to the hiding place and to the child, respectively, thus decided the position of the mirror. However, the mirror surface was proportionally the same size as the monitor, taking into account its position further away from the child. Considerations also had to be made to distinguish pre-recorded video from live video, from the point of view of the child, while keeping the visual information about the hiding constant. Arrangements were made to enhance features of pre-recorded film. First the attention catcher were animated pictures instead of the hand puppets in the other conditions, secondly, the assumption was that the difference in background (blue colored curtains) and a different person with different clothes (however, only torso and hands visible) would be enough for the child to perceive the pre-recorded video as different from the situation here and now. This of course supposes a balance between obtaining the feature of the medium and keeping the higher order iconicity equal.

### The Monitor and the Video Trials

The monitor was identically placed in both live video and pre-recorded video. The monitor fitted the opening in the occluder so that the directionality for perceiving the hiding event was kept constant with the direct perception trials. In live video, as opposed to recorded video, the hiding was depicted as occurring (and actually did occur) at the identical physical space (B) as in the direct perception trials. In the shifts between conditions, a shutter was used to conceal the installation of the monitor, and the children seemed to react with surprise and joy as they encountered the screen when the shutter was removed again. They thus seem to have distinguished the monitor in both live video and recorded video. Furthermore, the monitor was within reach of the child, and several children touched the screen and some also tried to “click”, “sweep” or “scroll” on its surface as if trying out a touch screen. The children frequently commented on, and pointed at, the monitor. All in all, we take these actions as indications that the children did not mistake the video projections for a window-like perception. Moreover, no child was insistent in their “clicking” or “scrolling” the monitor’s surface. When there was no response from the medium the children changed behavior to pointing without touching. We take this as an indication that the children differentiated the media object (Liben, 1999). Remarkably, in the direct perception condition, no child tried to reach through the opening to grab the cups; rather they wanted to walk around the barrier and enter the space from around the corner. No clear attempts of reaching through the monitor in live video and recorded video were observed.

The children were not trained in live video interventions in advance (Troseth, 2003) and were thus using live video as an information resource to guide a search as in this game for the first time. Some of the children were successful in using the information from live video not merely on their first trials, but repeatedly in later trials. Hence perseveration errors did not seem to interfere with their choices in live video (Schmidt and Anderson, 2002; Suddendorf, 2003). The results convincingly showed that the children benefited significantly less from recorded video than from live video in their subsequent retrievals. At the same time, we interpret the children’s willingness to make choices, and their disappointment when picking the cup with no token underneath, as a testimony that they nevertheless were motivated to retrieve the token. Taken together, the different results in recorded video and live video indicate that the children benefited from the perception of continuity in live video but lacked the alternative to bridge meanings by way of sign relations in recorded video. If the participants instead had relied on the convention of sign relations – where one thing says something about another thing although they are not perceived as identical – we should expect more similar result in recorded video as in live video.

### The “Video Deficit” and the Mirror Image

The children in this study were significantly more successful in retrieving the token when they were informed by means of live video than by seeing the event in a mirror. This result is intriguing. The continuity from the mirror perception allowed for an unbroken perception of the cups from the hiding place to the retrieval. Moreover, in a variation of the mark test (Gallup, 1970; Amsterdam, 1972; Suddendorf et al., 2007) observed that 2-year-old children responded differently to the live video projection of their own bodies than to their mirror images. As expected the 2-year-old children showed self-directed behaviors when presented to their mirror image; however, when presented to their live video reflection it was not until the age of three that the children rated equivalently to the performance of the 2-years old in the mirror version of the task (Suddendorf et al., 2007). The asynchrony remained even when video projections and mirror images were made more alike (e.g., in size, symmetry, reducing opportunity to eye contact). Indeed, (Suddendorf et al., 2007) doubted that impoverished visual information in video could be the reason for the 1-year lag between recognition of video compared to mirrors as measured by children’s self-directed behavior toward markings. Suddendorf et al. (2007) argued that this difference in 2-years-old children’s responses adds to the studies suggesting a video deficit effect in young children’s learning from video demonstrations. This may be taken to imply that the mirror image is “closer” to real-world perception than live video.

Mirror images, however, cannot simply be equated with perceptual reality, as our study shows (see also Sonesson and Lenninger, 2015). Nevertheless, the proximate relation to the reflecting surface (note also the possibility of accompanying sounds), the perfect contingency to one’s own body movements, and the tolerance for subjectively adjusted viewing angles in a mirror, might be factors that enhance perception of self (and contiguity to “reality”) also among the youngest perceivers. In contrast, in our study these cues for contiguity to reality are of less help in the mirror condition. Rather, we assume that the uneasiness in using the enantiomorphic information, and the dislocation of the mirror in relation to the experimenter’s space (B) are factors that can obscure perception of contiguity. In order to compensate for this “blurred” contiguity, positing a sign relation to the mirror image could have been helpful. 2-year-olds, however, are only at the beginning of learning to make use of sign relations.

## Conclusion

Pictures and video clips are examples of semiotic resources that are also signs. Thus, prior to being perceived as a thing and an instance of visual layout in itself, the picture may be experienced as conveying the meaning of depicted objects or scenes. This duality of *meaning* forms the basic model of the concept of sign in semiotic theory. Observations on (at least) dual meanings in signs are also central to psychological studies of children’s meaning-making (Piaget, 1945; Bruner, 1966; DeLoache, 2004). In fact, psychology and semiotics have for a long time entertained a dialogue on meaning and meaning-making that has been mutually enriching for our overlapping research interests (Lenninger et al., 2015).

With the present paper we hope to continue this dialogue by pinpointing the relevance to developmental studies of two semiotically distinct factors in meaning-making, even when basically iconic signs are involved: iconicity and indexicality (e.g., contiguity). More precisely, we wanted to investigate whether the perception of contiguity facilitated the linking of one instance of meaning to another (from media to the real world) independently of meanings being conveyed by signs or not. Psychological studies (Piaget, 1945; DeLoache and Burns, 1994) suggest that at the age of 2 years children typically have not yet developed the competence to use picture signs as a guide for solving a task in the real world. However, empirical studies also show that much younger children may be able to extract relevant meaning from pictures by identifying visual objects and separate them from the real objects (DeLoache et al., 1979; Liben, 1999). This conundrum was the point of departure of our study.

The low probability for the children to find the token in the pre-recorded video condition or by means of observing the hiding via a mirror, as contrasted with the high probability of finding it in the live video condition, supports the assumption that 2-year-old children do not yet use the potential sign relation subsisting between the information gleaned from the media and the actual cups, but that they had to have recourse to the direct perception of contiguity in order to facilitate their retrieval of the right cup in live video.

In addition, the current study demonstrates that a mirror image cannot simply be taken to be equivalent to direct perception – although it can momentarily be mistaken as such. Whereas the cups are never out of sight in the mirror, the image is both inverted and dislocated and therefore disrupts contiguity between the “mirror cup” and the “real cups” (see further Sonesson and Lenninger, 2015).

This suggests that the discernment of continuity, or more generally contiguity, is an elementary step in the development of human meaning-making. In addition, to grasp the notion of a sign by way of using reference relations, one has to understand that a sign expression is differentiated from its referent, in time and/or in space. The significance of iconicity in picture perception cannot be understated, since in the first-place pictures draw their meaning from showing similarity to their depicted objects (Sonesson, 1989; Simcock and DeLoache, 2006).

In school and at home, in many parts of the world, pictures and films are used for entertainment, but also as pedagogical implements for engaging children to learn about the real world. Pictures and films can be used to communicate instructions, or to offer information that helps someone to learn new facts about the world. More needs to be known, however, about how, and who, these media actually help. Recent studies have highlighted important aspects of this concern such as: children’s former experiences of the media (Troseth, 2003), contingent interactions with media devices (DeLoache et al., 1998; Lauricella et al., 2010; Kirkorian et al., 2016), social contingency expressed in the media communication (Strouse and Troseth, 2008; Strouse et al., 2018) the impact of the social situation in which the medium is experienced (Pempek et al., 2011; Strouse and Troseth, 2014; Choi and Krikorian, 2016; Kirkorian et al., 2016; Strouse et al., 2018) memory (Choi et al., 2018) and variations in iconicity (DeLoache and Burns, 1994; DeLoache, 2000; Simcock and DeLoache, 2006).

In the present study, the role of indexicality in understanding visual information from iconic media (e.g., video, mirror) is emphasized. Thus, it adds the semiotically informed concept of indexicality as a factor to understand pre-cursors to the development of sign use and young children’s perception of iconic media. The present study may be taken to suggest that the extent to which the continuity of ordinary perception is implemented in the experiment enters as a partially independent factor in the understanding of different iconically dominant semiotic resources.

## Data Availability Statement

All datasets generated for this study are included in the article/Supplementary Material.

## Ethics Statement

The studies involving human participants were reviewed and approved by the Regionala Etikprövningsnämnden i Lund. Written informed consent to participate in this study was provided by the participants’ legal guardian/next of kin.

## Author Contributions

On the basis on an original proposal formulated by GS, all authors contributed to the development, conception and design of the study. SL and TP piloted the study and organized the database. JW performed the statistical analysis and wrote sections of the manuscript (statistics). SL wrote the first drafts of the manuscript. All authors contributed to the manuscript revision, read and approved the submitted version.

## Conflict of Interest

The authors declare that the research was conducted in the absence of any commercial or financial relationships that could be construed as a potential conflict of interest.

## Footnotes

1 It will be noted that this sign definition, originally formulated in Sonesson (1989), does not require intention, in the sense of purpose, but certainly in the phenomenological sense of consciousness, since only in the presence of the latter will there be any differentiation as well as an asymmetry between expression and content. Note also (de Saussure, 1968 [1916]), and the tripartite division of the sign into Representamen (that which represents something), Object (that which is represented) and Interpretant (that which takes something to be representing something [else]) (Peirce, 1931–1958).
