# Supplementary material for: Mirror, Peephole and Video – The Role of Contiguity in Children’s Perception of Reference in Iconic Signs
Source: Front Psychol. 2020 Jul 14;11:1622. doi: 10.3389/fpsyg.2020.01622 (PMC7371794; doi:10.3389/fpsyg.2020.01622)
Supplement: Supplementary file 2 [file Data_Sheet_2.PDF]

```
mirror=read.table("mirror.csv",header=T,sep=",",stringsAsFactors=F)

# 1. Legend
# child: child ID
# gender: child gender (boy or girl)
# trial: trial number with the experiment (0 = first trial; 23 = last trial)
# block: experimental block (0 = first block (direct perception); 1 and 2 are second and third block)
# blocktrial: trial number within a block (0 = first trial; 1 = eighth trial)
# condition: (base = direct perception; stream = live video; pre-rec = recorded video; mirror = mirror)
# target: location of the target cup (left or right)
# choice: the cup that was selected (left or right)
# correct (0 = incorrect; 1 = correct)

# 2. Datafile modification
mirror$condition=factor(mirror$condition,levels=c("base","stream","pre-rec","mirror"))

# 3. Descriptive statistics (proportions correct)
tapply(mirror[,"correct"],list(mirror[,"condition"],mirror[,"block"]),mean)

# 4. Analysis: model comparison and posthoc contrasts
mod0=lme4::glmer(correct~1+(1|child),mirror,family="binomial")
mod1=lme4::glmer(correct~trial+(trial|child),mirror,family="binomial") # Boundary warning.
mod2=lme4::glmer(correct~-1+condition+(1|child),mirror,family="binomial")
anova(mod0,mod1,mod2)

cm=matrix(c(1,-1,0,0,
            1,0,-1,0,
            1,0,0,-1,
            0,1,-1,0,
            0,1,0,-1,
            0,0,1,-1),byrow=T,ncol=4)
rownames(cm)=c("base-stream","base-prerec","base-mirror","stream-prerec","stream-mirror","prerec-mirror")
summary(multcomp::glht(mod2,linfct=cm))

# 5. Barplot (Figure 6 in the publication)
par(mar=c(5,5,3,2));par(cex.lab=1.6)
barplot(plogis(coef(summary(mod2))[,1]),
   ylim=c(0,1),names=NA,cex.names=2.5,
   border=NA,ylab="Predicted proportion correct",axes=F)
axis(side=2,at=c(0,0.25,0.50,0.75,1))
mtext(text=c("direct\nperception","live\nvideo","prerecorded\nvideo","mirror"),at=c(0.7,1.9,3.1,4.3),side=1,padj=1,
line=0,cex=1.3)
segments(x0=c(0.7,1.9,3.1,4.3),y0=plogis(coef(summary(mod2))[,1]-1.96*coef(summary(mod2))[,2]),
         x1=c(0.7,1.9,3.1,4.3),y1=plogis(coef(summary(mod2))[,1]+1.96*coef(summary(mod2))[,2]))
```
